# The Effect of a 12-Week Physical Exercise Program on Glycemic Indices in Adults at Community Wellness Services, Primary Health Care Corporation, Qatar, in 2023

**DOI:** 10.7759/cureus.79720

**Published:** 2025-02-26

**Authors:** A. Alyafei, Salam M Alkiswani, Hebah O M. Rbabah, Sara T Al Abdulla, Senda Amdouni

**Affiliations:** 1 Wellness Programs, Preventive Health, Primary Health Care Corporation, Doha, QAT

**Keywords:** fasting blood glucose (fbg), glycated hemoglobin a1c%, glycemic control, glycemic indices, physical exercise, type 2 diabetes mellitus

## Abstract

Background: The substantial rise in various risk factors for noncommunicable diseases in Qatar is well-documented, underscoring their contribution to premature morbidity and mortality. Physical exercise (PE) is pivotal, offering significant benefits for biochemical and anthropometric parameters while serving as a therapeutic and preventive measure. Community-based wellness centers within primary care provide a valuable opportunity to assess the impact of PE on different health aspects, including glycemic indices and anthropometric measurements.

Methods: This retrospective cohort study examines the data of adults who completed a 12-week PE in Primary Health Care Corporation (PHCC) wellness centers from 2021 to 2023. A total of 909 patient data sets were extracted from electronic medical records across the seven wellness centers at PHCC. Participants completed three moderate-intensity PE sessions of 60 minutes weekly over the 12 weeks. Eligible patient data were assessed by the availability of the hemoglobin A1c glycated (HbA1c) in percentage and fasting blood glucose (FBG) levels before and after the program, looking for the mean difference significance in the two parameters. Further analysis was done to correlate the sociodemographic and anthropometrics to the glycemic indices and consider the significance at p < 0.05.

Results: In an eligible cohort of 739 data sets, the mean age was 48.75 ±12.83 years, and 74.56% were women. The body mass index (BMI) preintervention was 31.15 ± 5.39 kg/m^2^, and the fat mass (FM) was 33.91 ± 8.42 kg, indicating obese patients. The majority of the data suggested that normoglycemic individuals were at the prediabetes stage (14.34%) and within the uncontrolled diabetes range (4.48%). The mean reduction in FBG post-PE was 0.18 mmol/L (95% CI = 0.016-0.401; p = 0.000; paired t*-*test). In contrast, the HbA1c% decreased by a mean of 0.050% (95% CI = 0.006-0.135), with a significant association favoring the PE (p = 0.035, paired t-test). Notably, 31.13% of 106 patients with prediabetes normalized their HbA1c% after PE, while 40% of uncontrolled patients with diabetes transitioned to a controlled glycemic state out of 35 cases. All the anthropometrics demonstrated a statistically significant relation in their mean difference, favoring the effect of PE, where weight (0.95 kg; 95% CI = 0.61-1.44), BMI (0.27 kg/m^2^; 95% CI = 0.23-0.49), and FM (1.4 kg; 95% CI = 0.94-1.7) also correlate with the change in glycemic indices.

Conclusion: The 12-week structured PE intervention at PHCC wellness centers elicited a profound and statistically significant amelioration in glycemic indices. The observed reductions substantiate the metabolic benefits of sustained PE, with notable implications for insulin sensitivity and glucose homeostasis. These results accentuate the indispensable role of exercise as a therapeutic and preventive modality, which supports the need for integrating such programs into primary healthcare frameworks.

## Introduction

The prevalence of noncommunicable diseases (NCDs), such as type 2 diabetes mellitus (T2DM) and obesity, is quite significant in Qatar and constitutes a major contributor to premature mortality and morbidity. This burden is closely linked to significant lifestyle changes within the Qatari population over the last few decades [1,2]. A healthy lifestyle, including physical exercise (PE), is one modality to prevent and manage diabetes in addition to regular pharmacological interventions at the primary healthcare level [3,4].

The literature discusses PE's effect on glycemic homeostasis, addressing its favorable impact on preventing and controlling glycemic indices among adults. Through their systematic review and meta-analysis, Liu et al. reported that the PE cardinal role profoundly benefits biochemical parameters, particularly glycemic control [5]. Chang et al. [6] conveyed the positive impact of PE on prediabetes and related metabolic variables, as concluded by Hu et al. [7] similarly. Other authors illustrated the possibility of reversing prediabetes to an expected average glycemic level using a structured PE program [8,9]. Besides its positive effect on prediabetes, Xue et al. confirmed that regular PE substantially improves glycemic control among diabetes patients with higher hemoglobin A1c glycated (HbA1c) levels, other anthropometric measurements, and other biochemical markers [10].

Evidence attributed PE's effect on glycemic indices to several factors, including sociodemographic and anthropometrics. Age looks to be an essential factor in facilitating the PE role, as Jia et al. addressed that the younger the age group, the more effective the influence of PE on glycemic control, a similar finding reported previously by Cuenca-García et al. [11,12]. Gender is another determined factor; men tend to engage in more prolonged or intense PE activities than women, reflected in better glycemic control, as Whipple MO and colleagues concluded [13].

We evaluated the correlation between anthropometrics, including weight, waist circumference (WC), body mass index (BMI), fat mass (FM), and glycemic control. The negative correlation illustrated that the lower the anthropometrics, the better the glycemic control [14].

The wellness program at the Primary Health Care Corporation (PHCC), a governmental institute that leads primary care provision in Qatar, is an initiative to support the population's adoption of healthier lifestyles through PE programs and other interventions. Seven wellness centers nationwide deliver PE programs, as part of the management plan, to adult clients referred to by family medicine or healthy lifestyle clinics. The centers provide pre- and post-PE assessments, including biochemical markers such as glycemic indices for all patients referred to them, as well as anthropometric measurements, making it an opportunity to assess the effect of PE.

This study aims to evaluate changes in glycemic indices, measured by HbA1c% and fasting blood glucose (FBG), among adults who completed a 12-week PE program at wellness centers in 2023. Additionally, it examines the relationship between sociodemographic factors, anthropometric measurements, and glycemic index changes.

## Materials and methods

Study design and setting

This retrospective study used data extracted from the Electronic Medical Records System (EMRS) of seven PHCC wellness centers between January 2022 and December 2023, which was subsequently analyzed for these centers.

Study population

The study included adults (≥18 years old) who completed a 12-week PE program at the seven PHCC wellness centers between January 2022 and December 2023.

Intervention

The intervention in this study was a 12-week PE program conducted at the seven wellness centers within the primary care centers. The PE program consisted of three weekly 60-minute sessions of moderate-intensity exercise, maintaining a heart rate above 70% of the patient’s maximum heart rate. Intensity was monitored using heart rate tracking during sessions, and all exercises were supervised by certified fitness professionals to ensure standardization and consistency. The PE program was supervised and mixed with aerobic exercises, resistance training against weight, and warm-up, workout, and cooling phases. Exercise adherence was monitored through attendance records in the EMRS, with participants required to complete at least 85% of scheduled sessions for inclusion in the final analysis. All participants underwent pre- and postintervention assessments, including glycemic indices and anthropometric measurements.

Sampling strategy

A consecutive sampling method was employed, including all eligible patients who completed the program and had pre- and postintervention data available. This confirmed that all eligible patient data within the timeframe were included without predefined selection criteria.

Exclusion criteria

To ensure the reliability and validity of the findings, patients with no completed data set, erroneous data entries, or missing key data points relevant to the study objectives were excluded. Furthermore, patients taking weight management medications and antihyperglycemic agents, such as metformin, insulin, or medical diet regimens, were excluded if these treatments were initiated or altered within three months before the intervention.

Data collection

Data were extracted from PHCC EMRS of patients who completed the 12-week PE program and had relevant blood test results available. Age (in completed years) and gender (male or female) were recorded. Glycemic indices included FBG (mmol/L) and HbA1c (%). Anthropometric measurements included weight (kg), WC (cm), BMI (kg/m²), and FM (kg). The deidentified research data were securely stored in a password-protected Microsoft Excel® file (Microsoft Corporation, Redmond, WA), accessible only to authorized research team members.

Quality measures

The PHCC Institutional Review Board (IRB) approved the study, ensuring compliance with ethical guidelines. To maintain participant confidentiality, all data were unidentified. A standardized, IRB-approved data extraction protocol was developed to identify and record data from EMRS systematically. The research team, including the principal investigator, underwent comprehensive training to ensure consistency and accuracy. Multiple team members performed regular reliability checks to verify the consistency of patient record reviews, and discrepancies were resolved through consensus or further review by the principal investigator. Only complete and comparable data sets were included in the analysis, and routine audits and validation procedures cross-referenced extracted data with EMRS source documents to ensure accuracy and protocol compliance.

All anthropometric measurements, including the body composition analysis using approved calibrated bioelectrical impedance analysis machines and standardized stadiometers (Detecto Scale Inc, Brooklyn, NY), were taken according to the PHCC clinical practice guidelines, which were compatible with international recommendations. The BMI calculations and WC cutoffs followed World Health Organization guidelines [15]. The anthropometric measurements were taken by trained healthcare professionals at the PHCC wellness centers following standardized protocols. Measurements were conducted at two timepoints: preintervention (baseline) and postintervention (week 12).

Before the wellness center referral, all patients were directed to undergo a standard blood test, which includes glycemic indices of HbA1c% and FBG, as well as other biochemical markers. The blood tests were analyzed in PHCC-affiliated laboratories using validated biochemical assays.

The normoglycemic value for HbA1c% was less than 5.7%, while 5.7%-6.4% and more than 6.5% were considered prediabetes and diabetes individuals, respectively. In contrast, a value of more than 7.2% was considered uncontrolled T2DM regardless of age [16].

Data analysis

Descriptive statistics were used to summarize patients’ demographic and preintervention clinical characteristics. Categorical variables (e.g., age groups, and gender) were presented as frequencies and percentages, while continuous variables were summarized as means ± standard deviations. Moreover, we calculated and summarized the averages for weight, BMI, WC, FM, HbA1c%, and FBG. We further stratified the data based on HbA1c% to determine the classifications of normoglycemic individuals, those with prediabetes, and individuals with uncontrolled diabetes.

Mean differences in glycemic profiles were calculated, and their statistical significance was evaluated using the paired t-test. Box plots were generated to visualize pre- and postintervention glycemic profile distributions. The percentage of reversing HbA1c% to normal ranges for prediabetes and uncontrolled diabetes was calculated. Additionally, the relationship between demographic characteristics of age and gender and anthropometric measurements with glycemic changes was further investigated using a correlation test. All statistical analyses were conducted using IBM Statistical Package for the Social Sciences Statistics for Windows, version 26 (Released 2019; IBM Corp., Armonk, NY), with a p value of less than 0.05 considered statistically significant.

## Results

Among 739 eligible participants, 81.30% out of 909 had an overall mean age of 48.75 ± 12.83 years, reflecting a diverse age range across all patients. Almost three-quarters of the patient data (74.56%) were for women, as shown in Table 1.

**Table 1:** The characteristics of the patients before the 12-week physical exercise (n = 739) SD: standard deviation; WC: waist circumference; BMI: body mass index; HbA1c: hemoglobin A1c glycated; FBG: fasting blood glucose

Category	n (%)	Mean ± SD
Age categories (completed years)		
18-30	73 (9.88%)	24.52 ± 3.45
31-65	609 (82.41%)	49.57 ± 8.86
˃65	57 (7.71%)	70.98 ± 4.95
Total	739 (100%)	48.75 ± 12.83
Gender		
Female	551 (74.56%)	-
Male	188 (25.44%)	-
Total	739 (100%)	-
Anthropometric measurements		
Weight (kg)	-	80.00 ± 14.71
WC in males (cm)	-	103.98 ± 12.51
WC in females (cm)	-	98.52 ± 13.04
BMI (kg/m²)	-	31.15 ± 5.39
Fat mass (kg)	-	33.91 ± 8.42
Glycemic indices		
HbA1c (%)	-	5.87 ± 0.89
FBG (mmol/L)	-	5.94 ± 1.20
Normoglycemic HbA1c <5.7%	600 (81.19%)	5.27 ± 0.366
Prediabetic HbA1c 5.7%-6.4%	106 (14.34%)	5.92 ± 0.194
Uncontrolled diabetes HbA1c ≥7.2	33 (4.48%)	8.29 ± 1.161
Total	739 (100%)	-

The mean body weight before the 12-week PE program was 80.00 ± 14.71 kg, and the mean WC was 103.98 ± 12.51 cm for men and 98.52 ± 13.04 for women reflecting differences in abdominal adiposity. The preintervention BMI was 31.15 ± 5.39 kg/m², and the mean FM was 33.91 ± 8.42 kg. The preintervention glycemic indices showed that the average for the HbA1c level was 5.87% ± 0.89%, while it was 5.94 ± 1.20 mmol/L for FBG. Most participants were normoglycemic, while 14.34% were classified as having prediabetes, and 4.48% had uncontrolled diabetes.

**Figure 1:**
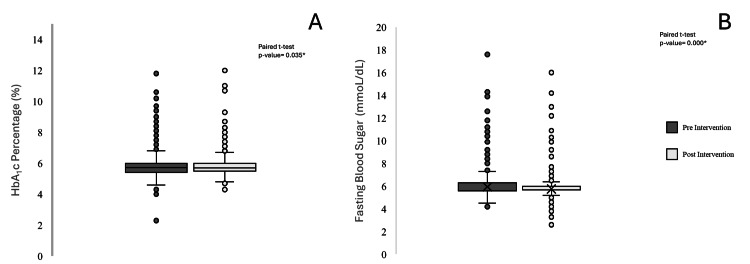
The effect of 12-week physical exercise on glycemic indices in adults at wellness services, PHCC. (A) Box plot of the mean difference between pre- and postintervention HbA1c percentage. (B) Box plot of the mean difference between pre- and postintervention fasting blood glucose (mmol/L). Paired t-test ^*^A paired t-test revealed a statistically significant reduction in HbA1c (%) and fasting blood sugar (mmol/dL) levels following the intervention (p < 0.05). Despite the presence of outliers, the observed decrease suggests a meaningful effect of the intervention on glycemic control Statistical significance was evaluated using the paired t-test HbA1c: hemoglobin A1c glycated; PHCC: Primary Health Care Corporation

When comparing glycemic indices before and after the 12-week PE program, the mean HbA1c level decreased from 5.87% to 5.82% postintervention. The paired t-test for HbA1c yielded a p value of 0.035, indicating that the reduction was statistically significant. In contrast, the mean FBG level decreased from 5.94 preintervention to 5.76 mmol/L postintervention. The paired t-test (p value of 0.000) confirms a significant FBG reduction following the intervention, which favors the effect of the exercise on improving the glycemic biochemical indicators, as illustrated by the box plot in Figure 1.

Table 2 demonstrates the effect of PE on reducing all anthropometric measurements following a 12-week exercise program. The mean difference between the pre- and post-PE programs for weight, WC for men and women, BMI, and FM was 0.95 kg, 0.99 cm, 2.34 cm, 0.27 kg/m^2^, and 1.42 kg, respectively. On assessing the statistical significance of the change in each variable of the anthropometric measurements, all anthropometric measures showed significant improvements, supporting the beneficial effects of PE, a paired t-test for the mean with a p value of <0.000.

**Table 2:** The effect of 12-week physical exercise on glycemic indices and anthropometric measurements Statistical significance was determined using a paired t-test, with a p value of <0.05 considered statistically significant HbA1c: hemoglobin A1c glycated; FBG: fasting blood glucose; WC: waist circumference; BMI: body mass index; SD: standard deviation

Parameter	Preintervention (mean ± SD)	Postintervention (mean ± SD)	Mean difference	p value
Glycemic indices				
FBG (mmol/L)	5.94 ± 1.20	5.76 ± 0.91	0.18	0.000
HbA1c (%)	5.87 ± 0.89	5.82 ± 0.73	0.05	0.035
Anthropometric measurements				
Weight (kg)	80.00 ± 14.71	79.05 ± 13.89	0.95	0.000
WC in males (cm)	103.98 ± 12.51	102.99 ± 12.22	0.99	0.010
WC in females (cm)	98.52 ± 13.04	96.18 ± 13.12	2.34	0.000
BMI (kg/m^2^)	31.15 ± 5.39	30.88 ± 5.09	0.27	0.000
Fat mass (kg)	33.91 ± 8.42	32.49 ± 7.35	1.42	0.000

Further analysis indicated that 33 of the 106 (31.0%) prediabetes individuals reversed their HbA1c% to the normal range of less than 5.7%. In comparison, 14 out of the 35 (40%) uncontrolled T2DM reached their controlled range of less than 7.2% of HbA1c% following the 12-week PE program.

Pearson’s correlation analysis assessed associations between glycemic improvements and sociodemographic/anthropometric factors. However, multivariate regression models were not applied (Table 3). Male patients might have a slightly more significant decrease in FBG than female patients, as indicated by a negative correlation (r = -0.207). The change in HbA1c shows minimal gender differences with a slight negative correlation of r = -0.050. Regarding age, a slightly negative correlation (r = -0.135) suggests that younger participants might experience a somewhat more significant improvement in FBG. Similarly, a marginally better improvement in HbA1c was observed for younger participants, as indicated by a negative correlation of r = -0.074.

**Table 3:** Correlation matrix between the changes in the glycemic indices and age, gender, and anthropometric measurements Pearson’s correlation coefficient (r) was performed to assess associations HbA1c: hemoglobin A1c glycated; FBG: fasting blood glucose; WC: waist circumference; BMI: body mass index

Variables	Age	Gender	Difference in FBG	Difference in HbA1c	Difference in weight	Difference in WC	Difference in BMI	Difference in fat
Age (years)	1.00	-	-	-	-	-	-	-
Gender (M/F)	0.25	1.00	-	-	-	-	-	-
Difference in FBG	-0.13	-0.21	1.00	-	-	-	-	-
Difference in HbA1c	-0.07	-0.05	0.47	1.00	-	-	-	-
Difference in weight	0.11	0.01	0.02	0.04	1.00	-	-	-
Difference in WC	0.03	0.04	-0.01	0.05	0.35	1.00	-	-
Difference in BMI	0.06	-0.01	-0.01	0.02	0.68	0.49	1.00	-
Difference in fat	0.01	0.02	0.01	0.05	0.35	0.25	0.37	1.00

Regarding anthropometric measurements with the change in the glycemic indices, BMI was negatively correlated with changes in FBG and HbA1c, indicating that individuals with higher BMI might have experienced more significant improvements. FM reduction was associated with improved glycemic indices, suggesting a link between body fat loss and better glycemic control. WC demonstrated a moderate negative correlation with changes in FBG and HbA1c, indicating that reducing central obesity may contribute to better glycemic regulation.

## Discussion

This study's objectives were to evaluate changes in glycemic indices, measured by HbA1c% and FBG, among adults who completed a 12-week PE program at wellness centers in 2023. Additionally, the relationship between sociodemographic and anthropometric measurements and changes in the glycemic indices was assessed.

This study confirms the PE program's positive influence in inducing a noticeable change in the glycemic indices after 12 weeks of intervention. Such effect of the PE was previously reported by Wang et al. using similar study designs and confirmed by others [17-19]. The role and mechanism of PE on glycemic homeostasis have been investigated thoroughly in the literature, suggesting multiple metabolic, cardiac, and other factors related to body composition that contribute to improving the glycemic indices [20].

The factors contributing to such a noticeable change in HbA1c% and FBG in this study could be age and gender. The study illustrated a relative negative correlation (r = -0.135) that the younger age could perform and sustain the PE intensity and practice, where the mean was 48.75 ± 12.83 years; a similar conclusion was demonstrated by Wing et al. Usually, younger age is associated with more engagement than older patients due to better mobility or fewer comorbidities [21]. In this study, although most of the sample studied were women (accounting for 74.56%), men exhibited a greater change in the glycemic indicators compared to women, indicated by a negative correlation of r = -0.207, which aligns with comparable results reported by Reading and LaRose [22]. A possible explanation could be related to the physiological and anatomical differences and the barriers to more intense exercises shown among women [23].

The study found that 31% of the participants reversed their biochemical markers of HbA1c from the prediabetes range (5.7%-6.4%), an essential point from a preventive perspective. Jadhav et al. reported consistent findings [24].

This research confirmed the relationship between anthropometric measurements and changes in glycemic indices. All the anthropometric measurements demonstrated a significant mean difference after the 12-week PE program. Furthermore, the measurements correlated negatively with the change in glycemic control and anthropometrics. Likewise, Verma et al. evidenced the association between glycemic biomarkers and anthropometrics. Zouhal et al. confirmed this by addressing the influence of PE on different anthropometric measurements [25,26].

In the current study, all patients were engaged in mixed 60-minute exercises, joining aerobic and resistance training three days a week. The evidence is inconsistent with which type and dose of exercise is more beneficial for enhancing the glycemic indices. Umpierre et al. addressed the importance of exercise volume and concluded that combined types of aerobic and resistance PE were effective. Gallardo-Gómez et al. address the need for PE dose to reach optimum effect on the glycemic markers. However, Huang et al. concluded that all aerobic exercises, combined aerobic and resistance training, and resistance training alone positively affected insulin resistance and glycemic control in prediabetes patients [27-29].

This study lacked a control group, which limits the ability to isolate the effects of PE from other potential influences such as dietary changes, medication adherence, or lifestyle modifications. Future studies should consider employing randomized controlled trial designs to establish causality with greater confidence.

Moreover, this study evaluated glycemic and anthropometric changes over 12 weeks; however, the long-term sustainability of these improvements remains uncertain. Future research should incorporate extended follow-up periods to determine whether the metabolic benefits of structured PE are maintained over time. Also, more objective tools should be included to overcome the individual variability of PE effort.

The weakness of the current study was that it relied on the available data from the institutional EMRS, with patient inclusion determined by data availability rather than randomization. The overrepresentation of female participants reflects PHCC data, which indicates that female participants were the dominant users of primary care services in Qatar. The female predominance may affect the generalizability of findings to male participants. Given known physiological differences in PE response between genders, future studies should employ stratified sampling or gender-balanced recruitment strategies for more robust subgroup analyses.

Regardless of standardized measurement policies and healthcare workers' training, variations in anthropometric assessments may still lead to measurement bias. Additionally, the study could not simultaneously eliminate other confounding lifestyle factors, such as dietary intake or PE outside of the wellness centers, which could interfere with glycemic outcomes. Furthermore, causality cannot be established; while marked glycemic indices were observed, such changes cannot be solely attributed to exercise.

On the positive side of this study, the findings were critical approaches for analyzing EMRS data, determining the program's effectiveness, and inspiring forthcoming studies from time and cost perspectives. Using real-community data was beneficial as it measured the changes induced by a 12-week PE program in glycemic controls and anthropometrics in a community setting.

While the study reported statistically significant differences in glycemic indices, the clinical relevance of these reductions should be interpreted with caution. Although the average HbA1c and FBG reductions of 0.05% and 0.18 mmol/L, respectively, were small, they may be important for individuals at risk of developing diabetes.

A standardized "dose-response" relationship for PE should be taken forward for further study. Though the intervention was delivered according to a defined protocol, some variations in the intensity and adherence to exercise could affect outcomes. To improve replicability and precision, future studies should seek to determine optimal PE dosing strategies leveraging wearable fitness trackers or metabolic monitoring.

Notably, 31% of individuals with prediabetes returned to normoglycemia, emphasizing that structured PE has a potentially preventive effect at the community level. This study raised the importance of addressing PE as a cornerstone part of the management plan for patients with NCDs, but further studies, such as clinical trials, would support and identify the effect of such PE, not only in terms of biochemical markers and anthropometrics but also in terms of overall quality of life.

## Conclusions

Results from this study highlighted the need to introduce structured exercise programs into primary healthcare settings to aid glycemic control. The maximum between-group effect size should not be viewed as a universal target but as a reference point. Healthcare providers should prescribe personalized PE interventions that are well tolerated and feasible for each individual. Routine monitoring of adherence and intensity should be integrated into diabetes prevention and management to ensure optimal outcomes. While the retrospective study design does not confirm causal inference, the study provides valuable real-world evidence supporting PE's integration into managing chronic diseases. The need for further research in the form of clinical trials would be essential.
